# Role of HYBID (Hyaluronan Binding Protein Involved in Hyaluronan Depolymerization), Alias KIAA1199/CEMIP, in Hyaluronan Degradation in Normal and Photoaged Skin

**DOI:** 10.3390/ijms20225804

**Published:** 2019-11-19

**Authors:** Hiroyuki Yoshida, Yasunori Okada

**Affiliations:** 1 Biological Science Research, Kao Corporation, 3-28, 5-chome, Kotobuki-cho, Odawara-shi, Kanagawa 250-0002, Japan; 2 Department of Pathophysiology for Locomotive and Neoplastic Diseases, Juntendo University Graduate School of Medicine, Tokyo 113-8421, Japan

**Keywords:** HYBID, KIAA1199/CEMIP, hyaluronan, hyaluronan degradation, hyaluronan synthase, skin, fibroblasts, extracellular matrix, photoaging, wrinkle, sagging

## Abstract

Photoaged skin is characterized clinically by apparent manifestations such as wrinkles and sagging, and histologically by an accumulation of abnormal elastin and a severe loss of collagen fibers in the dermis. Quantitative and qualitative alterations in elastin and collagens are considered to be responsible for the formation of wrinkles and sagging. However, since the integrity of elastin and collagen fibers in the dermis is maintained by their interactions with hyaluronan (HA) and a proteoglycan network structure, HA degradation may be the initial process, prior to the breakdown of the fibrillary components, leading to wrinkles and sagging in photoaged skin. We have recently discovered a new HA-degrading mechanism mediated by HYBID (hyaluronan binding protein involved in hyaluronan depolymerization), alias KIAA1199/CEMIP, in human skin fibroblasts, and examined the implication of HYBID for skin photoaging. In this review, we give an overview of the characteristics of HYBID and its prospective roles in HA turnover in normal skin and excessive HA degradation in photoaged skin. In addition, we describe our data on the inhibition of HYBID activity and expression by plant extracts in skin fibroblasts; and propose novel strategies to prevent or improve photoaging symptoms, such as skin wrinkling, by inhibition of HYBID-mediated HA degradation.

## 1. Introduction

The skin is a multifunctional organ that is continuously exposed to many biological and environmental factors. Therefore, to maintain cutaneous and global homeostasis, the skin is endowed with structural integrity and homeostatic adaptation mechanisms such as a cutaneous neuroendocrine system [1,2]. However, with increasing age, the integrity is gradually lost and adaptive responses decay [1,2]. Skin aging is accelerated by environmental stressors such as ultraviolet (UV) radiation, pollutants, and microbial insults [1,2]. Chronic UV irradiation disrupts normal structure of the skin and causes photoaging. Photoaged skin is clinically recognized by rough skin texture, uneven pigmentation, increased vascular fragility, telangiectasia, sebaceous-gland hyperplasia, fine and coarse wrinkles, and sagging [3,4,5,6,7,8,9]. Among them, deep wrinkles and sagging in exposed areas including the face are an important issue, not only in cosmetic science but also in dermatology. Many biochemical and histological studies on photoaged skin have demonstrated massive accumulation of aberrant elastic material, and disorganized and damaged collagen fibers in the dermis. The skin showing these alterations is referred to as ‘solar elastosis’ [4,5,6]. Such pathological changes in dermal fibrillary components are thought to result in the formation of wrinkles and sagging [3,4,5,10,11]. However, collagen and elastin fibers are incorporated into the network structures composed of hyaluronan (HA) and proteoglycans, such as versican, in the dermis [12]. Degradation of these network structures appears to be essential prior to breakdown of collagen and elastin fibers in the dermis [13]. Hyaluronidases (HYALs), such as HYAL2 and HYAL1, were long thought to be key enzymes for HA degradation [14]. However, short interfering RNA (siRNA)-mediated knockdown studies of these genes failed to change HA-degrading activity in skin fibroblasts, indicating that different molecule(s) are responsible for HA degradation [15]. Thus, we searched for new molecules related to HA degradation by microarray analysis, and found that KIAA1199 has a key role in the binding and depolymerization of HA in normal human skin fibroblasts [15] (see below for the details). We have named this molecule HYBID (HYaluronan Binding protein Involved in hyaluronan Depolymerization) [15,16], which is also called CEMIP (cell migration inducing protein) [17], and further investigated the role of HYBID in HA metabolism in photoaged skin [13,18].

In this review, we give an overview of the current knowledge about the molecular mechanism of HA degradation by HYBID in normal human skin fibroblasts. We then describe our findings that overexpression of HYBID and loss of HA in the superficial dermis, i.e., the papillary dermis, are related to the formation of photoaging skin symptoms such as skin wrinkling and sagging. Finally, we propose the hypothesis that inhibition of HYBID-mediated HA degradation would be a useful remedy to improve skin wrinkling.

## 2. Diverse Properties and Rapid Turnover of HA in the Skin

HA is a linear glycosaminoglycan (GAG), which is ubiquitously present as a major component of the extracellular matrix (ECM) in vertebrate tissues, composed of only two sugars, β(1,3)-linked-D-glucuronic acid and β(1,4)-linked-*N*-acetyl-d-glucosamine. HA not only contributes to hydration but also regulates cell proliferation and migration [19]. Although high concentrations of HA are maintained in many organs, approximately half of the body’s total HA is contained in the skin [20]. The current model of HA turnover in the whole body is as follows: HA is rapidly cleaved within tissues from large native molecules (1000–10,000 kDa) to intermediate size fragments (10–100 kDa) in the extracellular milieu [20]. Most fragments released from the ECM are drained into lymphatic vessels and catabolized within the lymph nodes [21]. The remaining HA fragments enter the circulation and are finally cleared—predominantly in the liver, kidney, and spleen [21]. Indeed, approximately one-third of the body’s total HA is turned over daily, and the skin is the largest determinant organ for HA replacement with a metabolic half-life of 1–1.5 days [21]. Thus, precise control of HA degradation and synthesis is required for this turnover, and is considered to balance tightly the quantity of high-molecular-weight HA within tissues.

## 3. Molecular Mechanism of HA Synthesis in Skin Fibroblasts

In 1996, the three human HA synthase genes (*HAS1*, *HAS2*, and *HAS3*) were identified. The deduced amino acid sequences show that the three HAS isoforms share a high degree of homology (64.9–78.4%) and contain the putative glycosyltransferase catalytic sites, seven putative membrane-spanning regions, and UDP (uridine diphosphate)-binding motifs [22]. Polymerization of HA is considered to occur on the inner face of the plasma membrane, and the product is extruded or translocated through the HAS protein complex to the extracellular space [22]. Among the three *HAS* genes, *HAS1* and *HAS2* were reported to be responsible for HA production in normal human skin fibroblasts [23].

## 4. HA Degradation Mediated by Newly Discovered HYBID in Skin Fibroblasts

As opposed to HA synthesis, the molecular mechanism of HA catabolism was controversial. One current model of HA degradation is that high-molecular-weight HA is captured in cell surfaces by CD44 (a receptor for HA), and first depolymerized by HYAL2 into intermediate size fragments. Then, the intermediate HA fragments are cleaved to oligosaccharides within cells by lysosomal HYAL1 with actions of β-*N*-acetyl-glucurosaminidase and β-glucuronidase [14]. Although normal human skin fibroblasts were found to have the ability to degrade exogenously added high-molecular-weight HA (>1000 kDa) to intermediate-sized fragments (10 kDa to 100 kDa), CD44, HYAL2, and HYAL1 were unlikely to be involved in the HA degradation for the following reasons [15]. First, skin fibroblasts expressed CD44 and HYAL2, but not HYAL1. Second, knockdown of CD44 and HYAL2 using siRNAs showed no effect on HA depolymerization. Since these data suggested the presence of a new HA-degrading mechanism independent of CD44 and HYAL2, or HYAL1, in skin fibroblasts, we carried out a comprehensive survey of candidate genes whose expression levels were paralleled with HA depolymerization activity, and found that knockdown of *KIAA1199*, which was originally reported as a deafness gene of unknown function [24], abrogates HA-degrading activity in normal human skin fibroblasts [15]. We also provided the evidence that transfection of *KIAA1199* cDNA into cells (HEK293 and COS-7 cells) endows the ability to degrade HA, and showed that dermal fibroblasts in normal human skin express *KIAA1199* by immunohistochemistry and in situ hybridization [15]. Altogether, our data strongly suggest that KIAA1199 expressed by dermal fibroblasts has a key role in HA degradation in normal skin, and we have named this molecule HYBID [15,16].

Transmembrane protein 2 (TMEM2), a type II transmembrane protein with sequence similarities to KIAA1199, was recently reported to be a cell-surface hyaluronidase in mouse organs [25]. However, the evidence for HA degradation by TMEM2 was obtained in TMEM2-overexpressing cells by transfection with the gene. Importantly, although normal human skin fibroblasts express both TMEM2 and HYBID/KIAA1199, knockdown of TMEM2 by siRNAs did not abrogate HA degradation [26]. Therefore, little evidence is available for the direct involvement of TMEM2 in HA degradation in human cells such as skin fibroblasts.

## 5. Characteristics of HYBID-Mediated HA Degradation

### 5.1. Molecular Function of HYBID Protein

**Figure 1:**
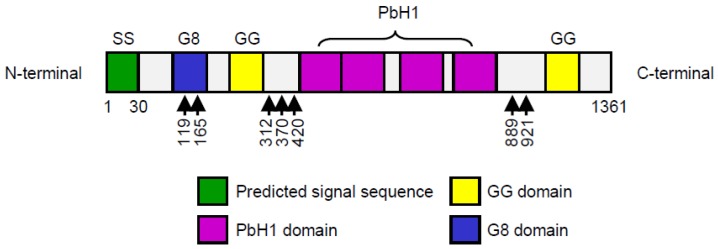
Structure of full-length HYBID (hyaluronan binding protein involved in hyaluronan depolymerization, also known as KIAA1199). Functional domains of HYBID are indicated as follows: SS (signal sequence), predicted N-terminal signal sequence; G8, G8 domain; GG, GG domain; PbH1 (parallel β-helix repeats), PbH1 domain. The predicted *N*-glycosylation sites are shown by arrows. Numbers indicate the positions of the residues relative to the N-terminus of the full-length HYBID.

*HYBID* (*KIAA1199*) is widely expressed in normal human organs including the brain, pancreas, lung, ovary, and testis, but is negligibly expressed in the liver, spleen, and kidney [27]. The cDNA contains a predicted 4083 bp open-reading frame encoding a protein with a molecular weight of 153 kDa (1361 amino acids) (Figure 1). The N-terminal portion of 30 amino acids functions as a signal sequence, which is required for proper translocation and HA depolymerization [28]. The HYBID (KIAA1199) protein has one G8 domain that contains eight conserved Gly residues in five β-strand pairs, two GG domains consisting of two well-conserved Gly residues [29,30], seven predicted *N*-glycosylation sites [31], and four PbH1 (parallel β-helix repeats) domains [31] (Figure 1). The function of the GG domain remains unclear at present, although mutations of the ARG^187^ residue (R187H and R187C) located in the GG domain reduce HA-degrading activity [15]. The G8 domain and the PbH1 domain are predicted to have roles in extracellular ligand binding [30] and polysaccharide hydrolysis [31], respectively.

### 5.2. Cellular Mechanism of HYBID-Mediated HA Degradation

**Figure 2:**
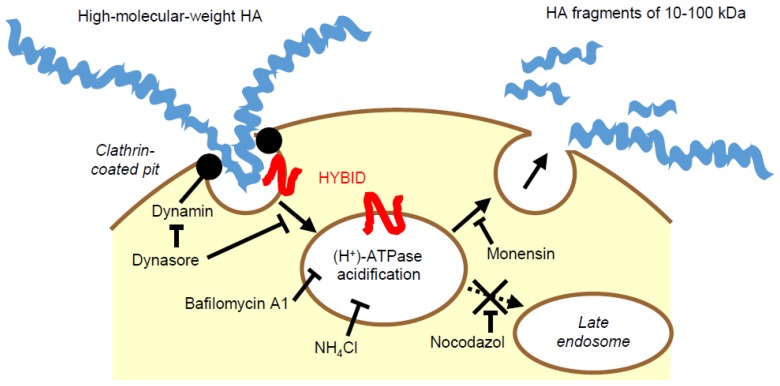
Schematic representation of our hypothesis on the HYBID-mediated hyaluronan (HA) degradation. High-molecular-weight HA is endocytosed to clathrin-coated vesicles, and cleaved to lower-molecular-weight HA fragments by the action of HYBID in acidic compartments. Then, the fragments are released extracellularly without intracytoplasmic accumulation.

HEK293 cells stably expressing HYBID (HYBID/HEK293 cells) degraded HA species with various molecular sizes in an endo-β-*N*-acetylglucosaminidase-dependent manner, but not other GAGs, including chondroitin sulfate A, C, and D, dermatan sulfate, heparin, or heparan sulfate, suggesting that the HYBID-mediated depolymerizing mechanism is specific to HA [15]. In addition, HYBID-mediated HA depolymerization is considered to occur through rapid vesicle endocytosis via the clathrin-coated pit pathway and recycling without intracytoplasmic accumulation or digestion in lysosomes according to the following findings [15] (Figure 2): (1) HA degradation was decreased in HYBID/HEK293 cells by knocking down the clathrin heavy chain (CHC) and α-adaptin subunit of AP-2, an adaptor protein complex, which is a major component of clathrin coats; (2) Treatment of HYBID/HEK293 cells with several pharmacological inhibitors for receptor recycling, endosome-lysosome system acidification, a vacuolar (H^+^)-ATPase (proton-pumping ATPase), or dynamin, abolished HA degradation, showed that HA is degraded in acidic compartments before endosome–lysosome fusion such as early endosomes or clathrin-coated vesicles; (3) Double immunostaining of HYBID and CHC in HYBID/HEK293 cells demonstrated that signals of both molecules are detected in a vesicular pattern and HYBID is localized closely to CHC in the peripheral vesicles of the cells; and (4) Fluorescence-labeled high-molecular-weight HA exogenously added to HYBID/HEK293 cells was observed transiently in the peripheral vesicles without intracytoplasmic accumulation. These observations of HYBID-mediated HA degradation match the current concept of the turnover of total-body HA: The initial HA degradation from large molecules to intermediate-sized fragments occurs in the extracellular milieu within tissues such as the skin, and most HA fragments are released from the ECM and transported to lymphatic vessels and bloodstream for further degradation and final clearance.

### 5.3. Characterization of Murine Homologue (mHybid) of Human HYBID

A murine homologue (*mHybid*) of human HYBID was cloned and reported to be expressed in mouse tissues [24]. The overall homology of the coding regions between mHybid and human HYBID proteins is 91% identical, and four PbH1 domains are completely conserved [28]. As expected, HEK293 cells transfected with *mHybid* cDNA can selectively catabolize HA into intermediate-sized fragments in an endo-β-*N*-acetylglucosaminidase-dependent manner via the clathrin-coated pit pathway [28]. Actually, *mHybid*-deficient mice show transient shorter long bones because of delayed endochondral ossification by accumulation of high-molecular-weight HA, which inhibits angiogenesis and osteoclast recruitment in the growth plate [32]. The mice would be useful in a study of the roles of this gene in physiological HA catabolism in vivo; and to investigate pathogenesis of HYBID and/or HA-linked human diseases [32,33,34].

## 6. Regulation of Expression of HYBID and HAS by Growth Factors in Skin Fibroblasts

Homeostasis of ECM components including HA is tightly controlled by synthesis and degradation in normal tissue, and it is altered under pathological conditions, in which growth factors and/or inflammatory cytokines are overproduced. Several growth factors, including TGF-β1 (transforming growth factor-β1), PDGF-BB (platelet-derived growth factor-BB), EGF (epidermal growth factor), and bFGF (basic fibroblast growth factor), are well-known to enhance HA production by upregulating HAS-mediated HA synthesis in normal human skin fibroblasts [23,35,36,37]. We have confirmed the data and further demonstrated that although TGF-β1 almost completely inhibits HYBID expression, PDGF-BB, bFGF, and EGF only mildly to moderately suppress the expression [16]. Our data have also provided important evidence that molecular sizes of newly synthesized HA are determined by the expression levels of HYBID, resulting in production of lower-molecular-weight HA fragments in PDGF-BB-, bFGF-, or EGF-treated fibroblasts, and high-molecular-weight HA formation in TGF-β1-stimulated fibroblasts [16]. The biological function of HA is dependent on its molecular size: high-molecular-weight HA is anti-inflammatory and anti-angiogenic [38], while low-molecular-weight HA fragments are potently pro-inflammatory and pro-angiogenic [39,40], and promote cell migration [41]. Therefore, in skin-wound healing, lower-molecular-weight HA species produced by the action of PDGF, bFGF, or EGF seem to be in accord with the tissue microenvironment for the inflammatory and proliferative phases, whereas high-molecular-weight HA production by TGF-β1 may be suitable for the remodeling phase [16].

## 7. Possible Involvement of HYBID-Mediated HA Degradation in Photoaging Skin Symptoms

### 7.1. Decreased HA in the Papillary Dermis of Photoaged Skin and Its Correlation with Photoaging Skin Symptoms

Alterations in HA during photoaging were controversial, and this was mainly because of the differences in the methods for normalizing HA data and/or temporal changes in HA amount, in association with the progression of photoaging [12,42,43]. In our study, we analyzed the amount and molecular weight of HA in paired biopsy specimens from control photoprotected skin (the inner arm) and photoaged (the corner of the eye) skin of the same Japanese females (age range, 65–72 years; mean age, 69.1 years), and found that both the amount and the molecular size of HA were decreased in photoaged skin compared to photoprotected skin (a 1.7-fold decrease in HA amount and 980 kDa versus 1840 kDa of peak HA molecular weight) [13]. These changes corresponded to a histological reduction of HA in the papillary dermis (superficial dermis with a depth of up to approximately 200 μm) in the photoaged skin [13]. In addition, the HA amount in the papillary dermis in the photoaged skin was negatively correlated with photoaging symptoms (skin wrinkling and sagging) [13]. Low-molecular-weight HA fragments are pro-inflammatory and pro-angiogenic [39,40], and promote cell migration [41], which is commonly observed in photoaged skin and may aggravate the skin damage [44]. Low-molecular-weight HA fragments and VEGF (vascular endothelial growth factor), which is induced by UV radiation in skin fibroblasts [45], may cooperatively contribute to excess angiogenesis [32], a characteristic histological feature of photoaged skin [44]. Altogether, our findings suggest that local HA reduction in the papillary dermis may be linked to the formation of skin wrinkles and sagging in photoaged skin.

### 7.2. Overexpression of HYBID in Photoaged Skin and Its Correlation with HA Amount in Papillary Dermis and Photoaging Skin Symptoms

**Figure 3:**
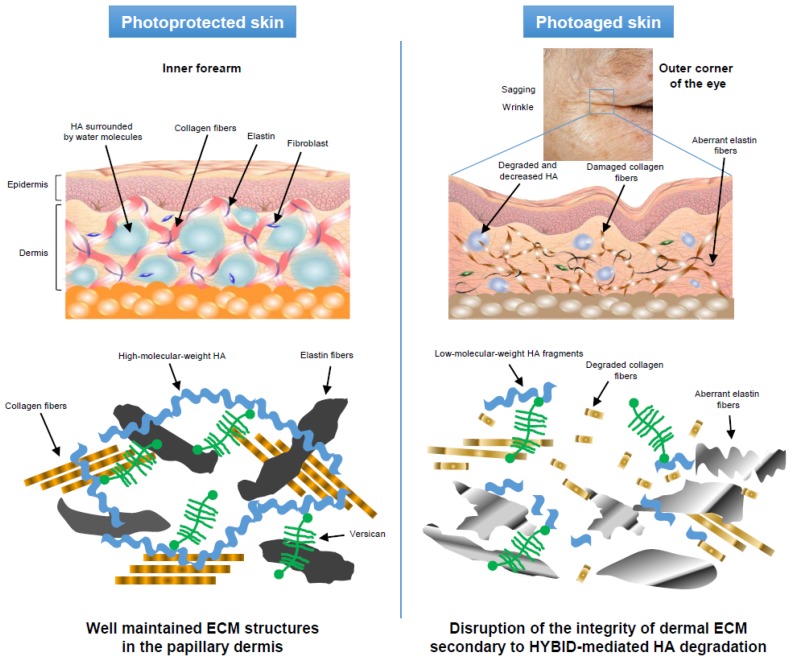
Overview of contribution of HYBID-mediated HA degradation and reduction in the papillary dermis to the photoaging skin symptoms. In the photoprotected skin (left panel), the integrity of the extracellular matrix (ECM) in the papillary dermis is well maintained, according to the balanced synthesis and degradation. HA is highly hydrophilic and surrounded with water molecules, producing a swelling pressure in the ECM and stabilizing skin structure. In the photoaged skin (right panel), HYBID-mediated HA degradation and reduction in the papillary dermis leads to reduced water binding properties and viscosity in the HA. This may contribute to disruption of the integrity of the dermal ECM by weakening the interaction with collagen and elastin fibers and promoting their degradation.

When the expression of *HYBID* and *HAS1/2* was compared, the photoaged skin showed increased expression of *HYBID* and decreased expression of *HAS1/2* [13]. In addition, the *HYBID* expression was reversely correlated with the HA amount in the papillary dermis of the photoaged skin, whereas no correlation was seen between the *HAS1/2* expression and the HA amount [13]. Importantly, *HYBID* expression was positively correlated with skin wrinkling and sagging [13]. The water-attracting property of HA produces a swelling pressure in the ECM, regulates ion flow, and stabilizes skin structure [46,47]. In addition, degradation and reduction of HA, which interacts with collagen and elastic fibers in the papillary dermis, may weaken the recoil capacity and tensile strength of these fibers. Taken together, HYBID-mediated HA degradation may cause a reduction in the size and amount of HA in the papillary dermis of photoaged skin, and these changes to the HA may be involved in photoaging symptoms such as skin wrinkling and sagging—possibly both—through the reduced water binding properties, viscosity, and turgidity in the HA, and by disruption of the integrity of the dermal ECM, including collagen and elastic fibers (Figure 3).

Although our studies suggested the importance of HYBID-mediated HA degradation in photoaged skin [13,46,47], reactive oxygen species (ROS) generated by UV radiation are known to randomly attack HA chains to generate HA fragments [48]. Therefore, our studies do not exclude the possibility that ROS are also implicated in HA depolymerization in photoaged skin. In addition, ROS induce gene expression of a variety of matrix metalloproteases (MMPs), which can in turn degrade many ECM components such collagens and versican, and thus these MMPs may also contribute to development of solar elastosis in photoaged skin [12,49].

### 7.3. Relationship of HA and HYBID Expression with Wrinkling of Photoaged Skin in Caucasian Females

Skin wrinkling is well-known to differ between ethnicities. For example, Caucasians have an earlier onset and greater degree of skin wrinkling than East Asians [50,51]. Thus, we examined the relationship of HA level and/or *HYBID* expression with skin wrinkling in Caucasian females (age range, 60–69 years; mean age, 62.2 years) by observing skin wrinkling of the outer corner of the eye and carrying out a biopsy of the same areas [18], and found that as in Japanese females [13], the expression levels of *HYBID* are positively correlated with the degree of wrinkling in Caucasian females, and that the HA amount in the papillary dermis is significantly reduced in skin with high-*HYBID* expression by dermal fibroblasts, compared to that with low-*HYBID* expression [18]. Our study suggests that HA reduction, mediated by HYBID in the papillary dermis of the photoaged skin at the eye corner, could be a common basis underlying wrinkle formation in both Japanese and Caucasian females aged over 60 years. However, since several factors including smoking, dietary habits, genetic background, sum of sun exposure, and skin structure are potential risk factors of wrinkling [52,53], further studies are needed to examine the roles of these factors in HYBID-mediated HA degradation and subsequent skin-wrinkle formation.

## 8. Screening of Agents that Inhibit the Expression and Activity of HYBID and Their Anti-Wrinkle Effects

In cosmetic science and dermatology, huge efforts have been made to develop agents that therapeutically prevent or improve photoaging symptoms. They may include melatonin, derivatives of vitamin D_3_ and lumisterol, and secosteroids, all of which are known to have protective effects on UV radiation-induced keratinocyte damage [54,55,56,57]. NS398, a COX2 inhibitor, was previously reported to downregulate *HYBID* mRNA expression in HP29 colon adenocarcinoma cells [58]. However, no information was available for cosmetic agents that can modulate HYBID expression or activity. Therefore, we screened extracts from plants that are traditionally used as herbal or folk medicines in Asia and/or Europe, and discovered that *Geranium thunbergii* (*G. thunbergii*) extract and *Sanguisorba officinalis* (*S. officinalis*) root extract completely inhibit HA degradation in HYBID/HEK293 cells and normal human skin fibroblasts [59,60]. These extracts not only inhibited HA-degrading activity of HYBID but also downregulated the *HYBID* gene expression, leading to production of high-molecular-weight HA [59,60]. Small-scale clinical trials of *G. thunbergii* extract and *S. officinalis* root extract as an anti-wrinkle agent were performed with Japanese females, who topically applied the test formulation containing each extract on one side of the outer eye-corner and the placebo formulation on the other side twice daily for eight weeks. As a result, *G. thunbergii* extract- and *S. officinalis* root extract-formulated lotions significantly enhanced the Ur/Uf (Immediate retraction/Final deformation) value (the ratio of elastic recovery to the total deformation), which is reported to negatively correlate with skin roughness [61,62,63], compared with the placebo formulation [59,60]. When a dermatologist evaluated the skin, the wrinkle scores of the side treated with the extract-formulated lotion were significantly lower than those of the other side treated with the placebo formulation [59,60]. Thus, these results suggest that inhibition of HYBID-mediated HA degradation by *G. thunbergii* extract and *S. officinalis* root extract would be a promising strategy for anti-wrinkle care.

Glucocorticoids are widely used to treat many inflammatory diseases including atopic or psoriatic skin, but the long-term use of glucocorticoids results in development of skin atrophy by reducing the ECM components including HA [64,65,66]. Although the effect of glucocorticoids on HYBID-mediated HA degradation is currently unclear, application of *G. thunbergii* extract or *S. officinalis* root extract might be expected to counteract glucocorticoid-induced skin atrophy by inducing production of high-molecular-weight HA.

## 9. Conclusions

By microarray analysis, we have identified a new molecule responsible for HA degradation independently of CD44 and HYAL enzymes [15]. This molecule was originally called KIAA1199, and is also called CEMIP by another group [17], but we named it HYBID [16]. HYBID has the ability to catabolize HA in an endo-β-*N*-acetylglucosaminase-dependent manner via the clathrin-coated pit pathway in normal skin fibroblasts [15]. This molecule determines the molecular size of newly synthesized HA depending on the expression level [16], and may play a key role in turnover of HA in the normal skin [15]. Photoaged skin displays prominent alterations in the reticular dermis, which is located under the papillary dermis, showing an accumulation of abnormal elastin and a severe loss of interstitial collagen fibers, and such quantitative and qualitative alterations of dermal ECM proteins have been proposed as candidates for wrinkle- and sagging-formation factors [3,4,5,10,11]. However, our recent studies suggest that HA reduction in the papillary dermis plays a central role in wrinkle and sagging formation in photoaged skin [13,18]. In contrast to photoprotected skin, synthesis and degradation of HA in the papillary dermis are unbalanced in favor of degradation by increased HYBID-mediated HA degradation and reduced HAS1/2-mediated HA synthesis in photoaged skin. In vitro and in vivo studies with *G. thunbergii* extract or *S. officinalis* root extract suggest the possibility that suppression of HYBID-mediated HA degradation by application of cosmetics or ointment containing inhibitors of HYBID would be a promising strategy to prevent or improve photoaging symptoms including skin wrinkling.

## Abbreviations

bFGF	basic fibroblast growth factor
CHC	clathrin heavy chain
ECM	extracellular matrix
EGF	epidermal growth factor
GAG	glycosaminoglycan
HA	hyaluronan
HAS	hyaluronan synthase
HYAL	hyaluronidase
HYBID	hyaluronan binding protein involved in hyaluronan depolymerization
MMP	matrix metalloprotease
PDGF	platelet-derived growth factor
ROS	reactive oxygen species
siRNA	short interfering RNA
TGF	transforming growth factor
TMEM2	transmembrane protein 2
UV	ultraviolet
VEGF	vascular endothelial growth factor
